# Dating the landscape evolution around the Chauvet-Pont d’Arc cave

**DOI:** 10.1038/s41598-021-88240-5

**Published:** 2021-04-26

**Authors:** Kim Genuite, Jean-Jacques Delannoy, Jean-Jacques Bahain, Marceau Gresse, Stéphane Jaillet, Anne Philippe, Edwige Pons-Branchu, André Revil, Pierre Voinchet

**Affiliations:** 1 Laboratoire Environnements Dynamiques et Territoires de Montagne, UMR 5204, CNRS, Savoie Mont Blanc University, Campus scientifique, Bat. Pôle Montagne, 73376 Le Bourget-du-Lac cedex, France; 2 Histoire Naturelle de L’Homme Préhistorique, UMR 7194, CNRS, MNHN, UPVD, 1, rue René Panhard, 75013 Paris, France; 3 Earthquake Research Institute, University of Tokyo, Tokyo, 158-8557 Japan; 4 Laboratoire de Mathématiques Jean Leray, Nantes University, 2, rue de la Houssinière, BP 92208, 44322 Nantes, France; 5 Laboratoire Des Sciences du Climat Et de L’Environnement, UMR 8515 CEA, CNRS, UVSQ, Orme des Merisiers, Bat 714, Chemin de Saint Aubin – RD 128, 91191 Gif sur Yvette cedex, France

**Keywords:** Archaeology, Geomorphology

## Abstract

The Chauvet cave (UNESCO World Heritage site, France) is located in the Ardèche Gorge, a unique physical and cultural landscape. Its setting within the gorge—overlooking a meander cutoff containing a natural arch called the Pont d’Arc—is also remarkable. Investigating possible associations between sites’ physical and cultural settings, chronologies of human occupation, and access conditions has become a major theme in archeological research. The present study aims to reconstruct the landscape of the Pont d'Arc meander cutoff during the Upper Paleolithic, when humans were present in the Chauvet Cave. We used uranium-series and electron spin resonance analyses to date the formation of the Pont d’Arc natural arch in the Combe d’Arc meander cutoff, near the Chauvet Cave. Results show that the meander became totally cutoff between 108 and 138 ka (95%). Hence, the natural arch formed before the Upper Paleolithic and the first known human presence in the Chauvet Cave, dated to 37 ka cal BP. These results allowed us to reconstruct a key part of the landscape surrounding the Chauvet Cave when it was being used by Upper-Paleolithic societies.

## Introduction

The fact that many major rock art sites are located in highly distinctive landscapes has led archeologists, ethnologists, and ethno-archeologists to explore potential links between landscape features and their symbolic usage^1–5^. The study of the physical context of the archaeological sites is of particular importance for expanding the interpretive horizons of the location of ancient societies and their interactions with living spaces. An increasing number of studies now focus on the restitution of site morphologies during their frequentation^6–11^. The main gaps in understanding concerns either the external geomorphological processes involved in the evolution of the external context of a site, the chrono-stratigraphic relationships between the deposits present on excavated sites and its surroundings, and the lack of integrated studies linking the information gathered on a site with its direct environment^12^.

**Figure 1:**
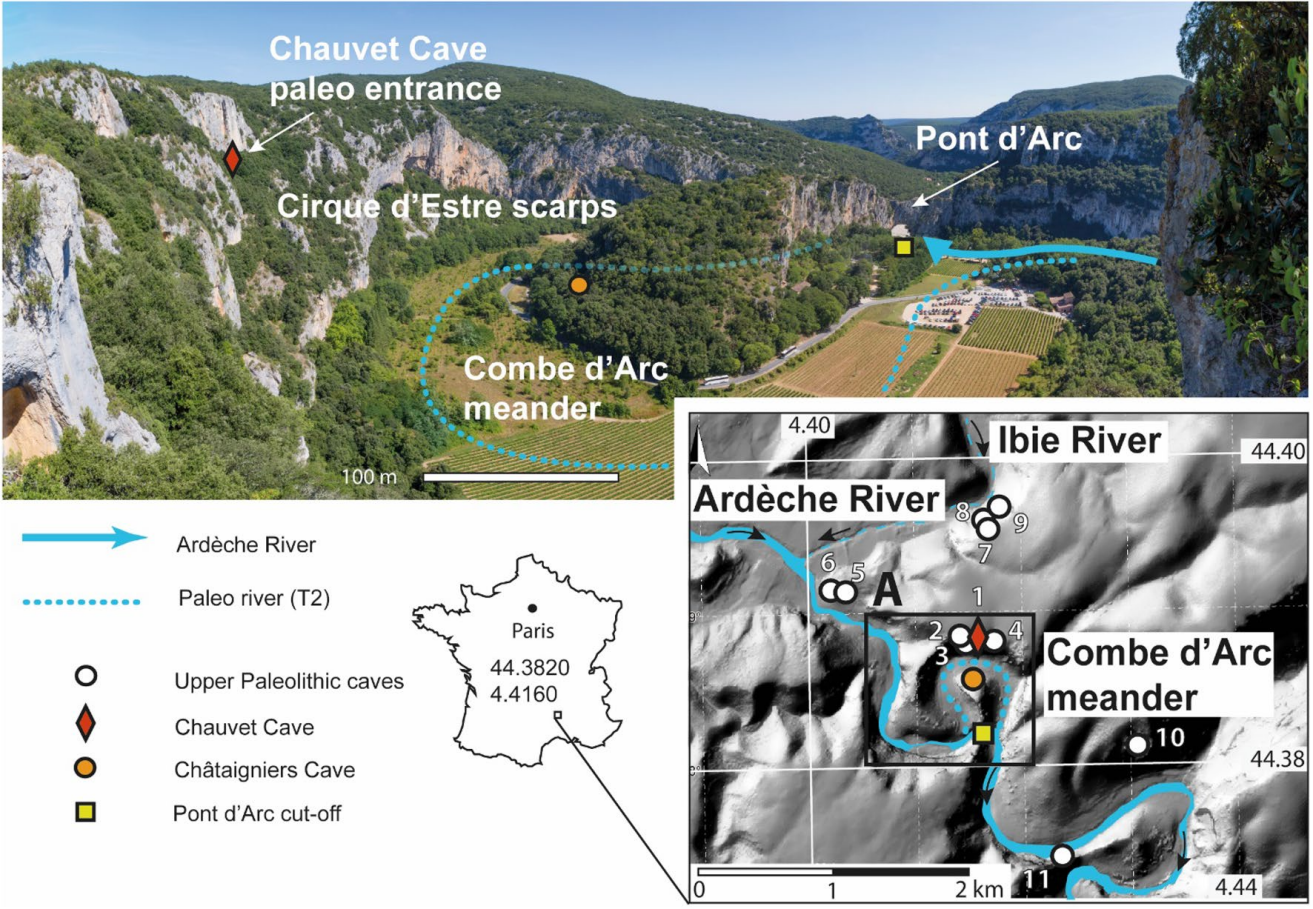
Location of the Chauvet Cave. The Chauvet Cave is in the Cirque d’Estre scarp, above the Combe d’Arc meander cutoff, which formed when the Ardèche River cut through the Pont d’Arc to form a natural arch. Several Upper-Paleolithic caves have been found in and around a stretch of the Ardèche Gorge, more-or-less centered round the Cirque d’Estre and Pont d’Arc. 1. Chauvet Cave, 2. Vacheresse Cave, 3. Planchard Cave, 4. Bergerie du Charmasson, 5. Mezelet, 6. Pc du Maquis, 7. Chasserou Cave, 8. Deroc Cave, 9. Fées Cave, 10. Baume du Bouchon, 11, Ebbou Cave. Photograph from: Jean-Jacques Delannoy.

The Chauvet cave benefited from such integrated geomorphological studies and cave morphologies could be reconstructed at different stages before, during and after the human frequentations^9^. The Chauvet cave Upper-Palaeolithic artwork was dated back to 37 ka cal. BP, and showed two main periods of frequentation at 37-33.5 ka cal. BP and 31–28 ka cal. BP respectively^13^. Its artwork characteristics and the antiquity of the radiocarbon ages made this cave a reference for the beginning of the Upper-Palaeolithic worldwide (UNESCO World Heritage). Exceptional artwork preservation is related with a series of rockfall events between 23 and 21 ka that blocked the Palaeolithic cave entrance, thereby preventing further access to the cave and preserving its paintings^14^. Such work contributed to highlight landscape evolution as an effective approach to contextualize archaeological research. However, previous studies have focused almost exclusively on the cave and its entrance, rather than its surroundings. Here we study the landscape evolution in the vicinity of the Chauvet Cave (Ardèche Gorges, France). In fact, the cave sides with numerous other Upper Paleolithic sites^15^ that lie around and inside the cliffs of the Cirque d'Estre, a meander cutoff that hosts a 30 m high limestone arch, called the Pont d’Arc, which spans the Ardèche River at the entrance to the Ardèche Gorges (Fig. 1). The concentration of Upper Paleolithic sites in such a specific landscape feature questions choice of the site frequentation by the prehistoric people^16–18^. An essential first step in conducting such analyses is to ascertain whether current features were already part of the landscape when a site was being used^9,11^. The present study aims to trace the evolution of this landscape in order to determine whether the meander cutoff and Pont d’Arc had already formed 37 ka cal BP, when the Chauvet cave was frequented by Upper Palaeolithic societies. The piercing of the natural arch allowed the river to bypass the meander^19^. We tried then to date the meander cutoff, as it would indicate when the natural arch formed.

**Figure 2:**
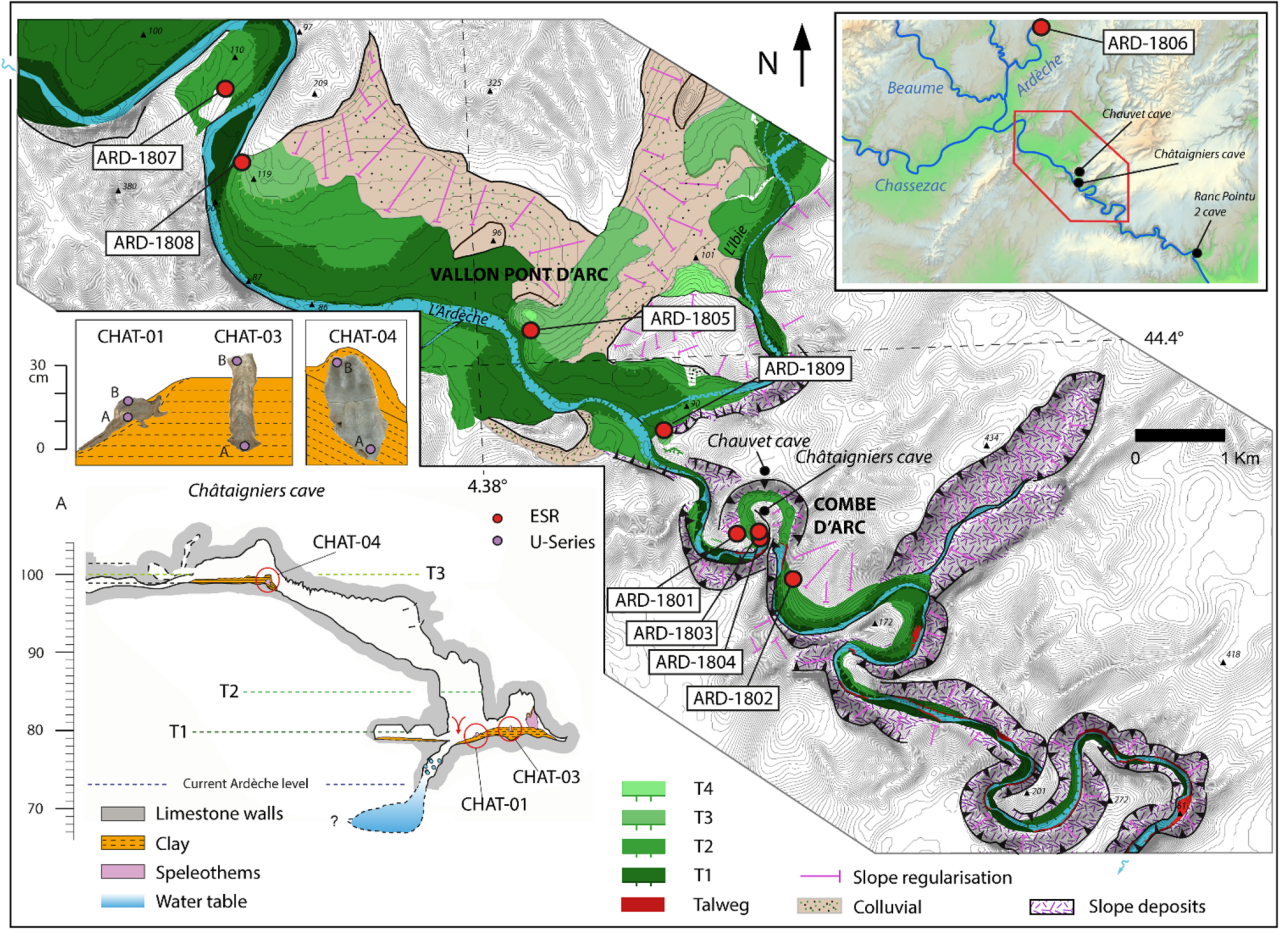
Geomorphological map of the Ardèche gorges entrance and sample location. Most of the ESR samples from the T3 and T2 levels were taken on the Vallon-Pont-d’Arc plain, in road cuttings (Photographs are reported in the Supplementary Fig. S2). The T1, T2 and T3 altitudes are reported on the Châtaigniers cave topography for comparison with the speleothem and clay deposits location. Geomorphological map from^19^, modified. Speleothem photographs from: Kim Genuite.

The first step was to carry out a geomorphological survey of the Ardèche gorges entrance. Because of its karstic context, the study focuses both the external morphological features (alluvial deposits) and sediments found in the proximate Châtaigniers cave system, located within the meander cutoff (Figs. 1, 2). The Châtaigniers cave was chosen because of its direct proximity with the Pont d’Arc, and for its range of altitude which corresponded both to the Pont d’Arc and the lower-level alluvial deposits heights^20^ (compared to the other surrounding caves), (Fig 2). We determined the surface and subsurface morphologies of the alluvial deposits by combining the results of topographical surveys with electrical resistivity data conducted inside the cutoff meander. Fieldwork investigations in the Ardèche canyon entrance led to determine three former river levels, which we labeled T1 (8 m above the current river base level), T2 (+15 m), and T3 (+30 m). Those observations were compared with the ones inside the Châtaigniers cave through a unified topographic and stratigraphic model. The comparison of the altitude of the deposits allowed their integration in a relative chronological model containing alternances of aggradation phases (coarse fluvial deposits in the river system and laminated clay deposits in the Châtaigniers cave) and erosion phases (river entrenchment and speleothem growth^20^. We then tried to date those respective phases.

Because of the quartz-rich component of the Ardèche river sediments^19,21,22^, we used paleodosimetric methods (Electron Spin Resonance – ESR) to date the river deposits^23–27^. In cave environment, radiometric analyses (Uranium series – U-series) are used to date the speleothems growth phases^28–31^. The combination of dating methods in both river and cave environment provides independent constrains that can be discussed through topographic and chronological correspondences^32–36^, potentially better constraining landscape evolution during the Quaternary period. The use of ESR on fluvial sediments and U-series in karst systems provides absolute constrains for landscape evolution studies in the Middle and Late Pleistocene.

Similar approaches combining relative and absolute dating of geomorphological objects have been used successfully at numerous archaeological sites^11,14,37,38^.

### Site setting

The Ardèche is one of the main tributaries of the Rhône river right bank and is located in the Massif Central eastern part (Cevennes mountain range). Its 2700 m^2^ catchment represents at least one third of the Cevennes Mountains, which reach 1700 m a.s.l, and extend from South-West to North-East with a mean altitude of 1200 m a.s.l. The Ardèche upper catchment is incised into plutonic and metamorphic formations. It then crosses sandstone Trias formation and finishes its course in the Rhône river incising the middle valley Jurassic sedimentary rocks and finally, the Cretaceous sedimentary rocks in the Ardèche gorges in the shape of entrenched meanders^21^. While the upper two third of the catchment are formed by the impervious rocks, the lower valleys incise carbonate rock shelves which are prone to karstification^39^. The Mediterranean conditions affecting the Ardèche watershed make its hydrological regime prone to flood events^40^. The Velay lakes constitute the closest reference palynological sequence for the middle to late Quaternary period^41^ and shows the impact of glacial and interglacial phases that may have affected the paleo-hydrological conditions in the Ardèche catchment.

The Vallon-Pont-d’Arc plain is a floodplain located at the entrance of the Ardèche gorges which contains stepped alluvial deposits^19,22^, (Fig. 2). The lower fluvial levels are organized parallel to the current rock talweg that crops out regularly in the river bed. The relative height above the river (RH) of the deposits is consistent throughout the middle and lower Ardèche valley^22^. The lower levels are the most visible in the current landscape and we focus here on the three lower ones (T1, T2 and T3) which have been characterized through geomorphological mapping and outcrops^19^, (Fig. 2). At the Ardèche canyon entrance, alluvial deposits are mainly composed of coarse elements (pebbles and cobbles ranging between 5 and 50 cm) with locally sand lens or sand beds (medium sands with stratigraphic alluvial features). They are organized as it follows: the T1 level is located at + 8 m (RH) and is characterised by large cobbles and boulders. Granitic and basaltic large cobbles (0.1–0.2 m) dominate the petrographic pattern^19,22,42^. The T1 characteristics is similar to the current alluvial plain^19^, Fig. 2). The T2 level (+ 15 m RH) is also well visible in the three main Ardèche plains. It is characterised by large cobbles (about 10 cm), and a low basaltic content^19^. The petrographic composition evolution of river terraces is impacted by both the late Miocene volcanism (Coirons lava) and late Pleistocene one (Bas-Vivarais) which occurred in the Ardèche catchment^43^. The current floodplain and T1 level composition are basalt rich, which is not the case for the T2 and T3 levels. Bas-Vivarais lava flows were deposited in the river talweg since between 80 and 30 ka and were subject to intense erosion^43^. The T3 level (+ 30 m RH) is characterized by slightly coarser cobbles than the T2 level (superior to 10 cm) and have an important spatial extent (Fig. 2). The sand matrix is also slightly more hardened although this was variable along the outcrops. In situ alteration was observed for about 20 % of the granite cobbles visible inside the T3 level at numerous outcrops^19^. This can be related with granite bisiallitisation into quartz sand^44,45^, as observed in other rivers in south of France^25,46^. The released quartz grains are millimetric to multi-millimetric in size.

The Châtaigniers cave is located in the vicinity of the cutoff meander and in the altitudinal range of the Pont d’Arc arch and the T1, T2 and T3 fluvial levels^20^. Trapped speleothems were visible at the corresponding T1 (+ 8 m RH) and T3 (+ 30 m RH) heights. They were in connection with laminated clay deposits linked with former water table levels in the cave.

## Results

### Geophysical prospection

**Figure 3:**
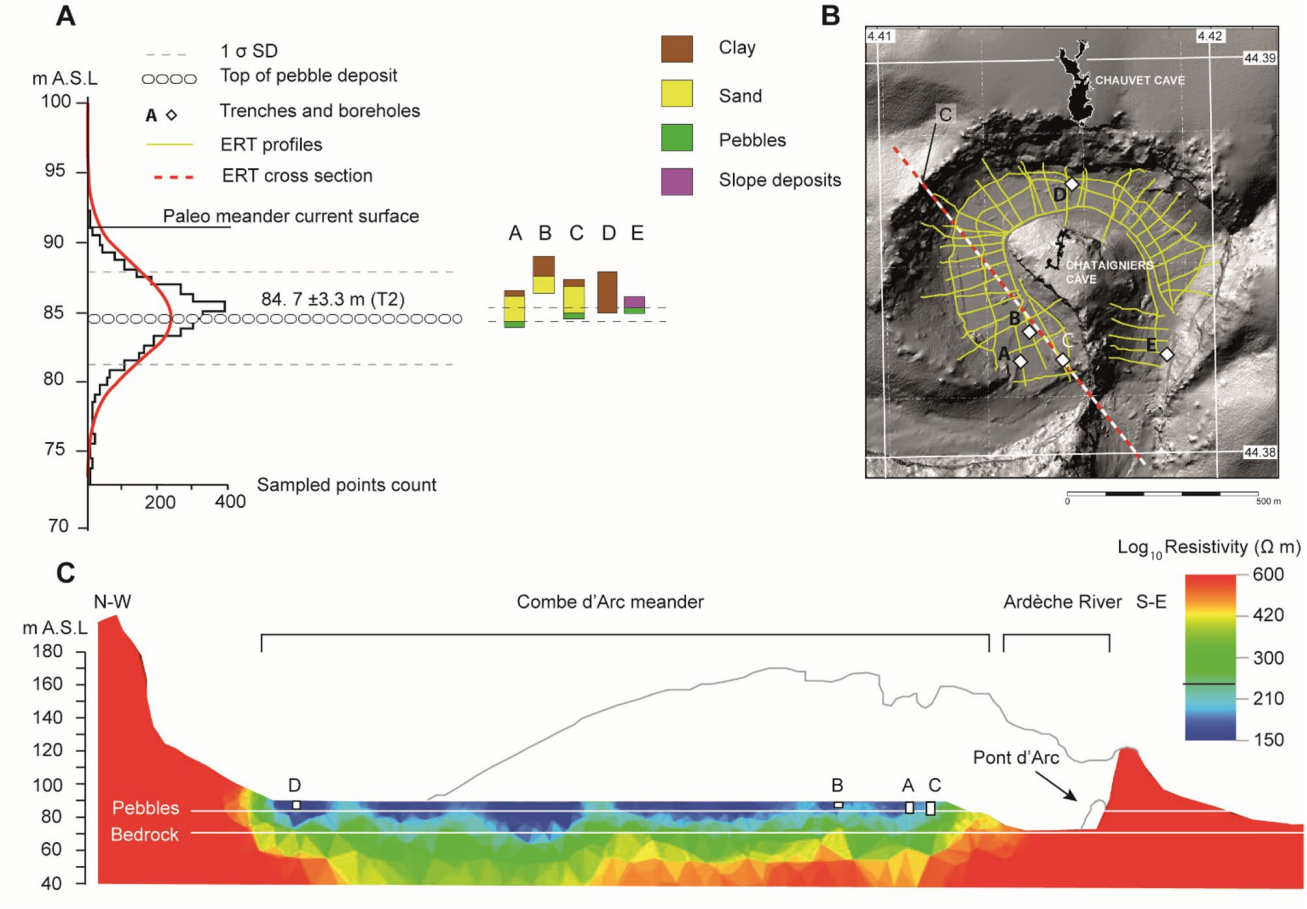
ERT survey inside the Combe d’Arc meander cutoff. Resistivity values for the meander sediments ranged from 150 to 600 Ωm. (**A**) Mean altitude of cutoff cobble layer. (**B**) Location of the ERT cross-sections. (**C**) Cross-section of the meander cutoff showing resistivity values.

For the +15 m alluvial deposit (T2), boreholes and trenches inside the Combe d’Arc (Fig. 3) revealed the presence of thin clay layers on top of layers of coarse sand and pebbles. The cobbles are linked to the last perennial flows through the meander before the cutoff became complete, while the clay is linked to flood events that occurred later in the cutoff meander^40^ (Fig. 3). ERT values of 250 Ωm were interpreted as corresponding to the top cobble deposit inside the meander. Plotting these values gave an altitude of 84.7 ±3.3 m A.S.L (±1 σ SD) across the entire meander, which is 4 to 7 m below the current surface (Fig. 3). This buried cobble-rich alluvial body indicates the last stage of the meander perennial activity, and thus represents the cutoff ending phase.

### U-series on speleothems

We obtained U-series ages for samples from three stalagmites (Table 1). CHAT-03 is a well-laminated, 20-cm-long stalagmite containing clay laminations that suggest that calcite growth was almost synchronous with the horizontal clay infilling. U-series ratios revealed high levels of detrital thorium that can be attributed to contamination during regular floods or water table rising within the cave, thereby suggesting that the speleothem grew concurrently with the deposition of the clay deposits in the cave. The ^230^Th/^232^Th ratio is relatively low, which is in agreement with probable contamination from detrital ^232^Th during the speleothem grow, which leads to more important age uncertainties, hence the reverse ages obtained for the speleothem growth (13.2 ±1,12 ka for the bottom and 15.7 ±2.9 ka for the top). The speleothem ages remain close to each other, and error ranges are slightly overlapped. Such age reversal is acceptable considering the time range aimed in the study. The CHAT-01 speleothem was sampled on the sides of the laminated clay infilling, in a lateral eroded part (Fig. 2). U-series delivered ages comprised between 7.25 ±1.95 and 2.34 ±1.54 ka. The relatively low ^230^Th/ ^232^Th ratio of CHAT-01 are also probably linked with detrital inputs (Supplementary Table S6). CHAT-04 is a 25-cm long stalagmite. The calcite appears to be free from clay deposits, suggesting that it grew well above the river base level, before being covered by clay sediments when the river level rose (Fig. 2). The good ^230^Th/ ^232^Th ratio show a probable absence of detrital contamination by high water table periods during the CHAT-04 speleothem growth (Supplementary Table S6). The top of the CHAT-04 speleothem gave a U-series age of 162.5 ±5 ka (CHAT-04B). This speleothem was later covered by clay sediments when the river level rose.

**Table 1:** U-series ages.

Ref	Sedimentary unit	Lithology	Method	Age
CHAT-04A	Speleothem	Calcite	U-series	364.24 ± 15.15
CHAT-04B	Speleothem	Calcite	U-series	162.46 ± 5.06
CHAT-03A	Speleothem	Calcite	U-series	15.660 ± 2.91
CHAT-03B	Speleothem	Calcite	U-series	13.170 ± 1.12
CHAT-01A	Speleothem	Calcite	U-series	7.25 ± 1.95
CHAT-01B	Speleothem	Calcite	U-series	2.34 ± 1.54

All ages are given in ka, with a 2 σ SD.

### ESR on fluvial quartz sediments

We used electron spin resonance (ESR) analyses to determine the ages of quartz-rich sediment samples from the +30 m (T3) and +15 m (T2) alluvial levels. The samples analyzed were taken inside the Combe d’Arc meander cutoff and upstream from the meander, mainly on the Vallon Pont d’Arc plain, which is immediately upstream from the entrance to the Ardèche Gorges (Fig. 2 and Supplementary Fig. S2).

Samples coming from the T3 level showed more important optical bleaching rates ranging between 60 and 90%, contrary to the T2 level which shows more common values around 50 %.

Preliminarly, Al and Ti-Li burial ages obtained on the six T3 level samples show an age repartition ranging between 179 and 511 ka. High bleaching rate affect all the samples of the T3 level. Samples taken in sandy matrix between pebbles show the highest bleaching rates (ARD 1801, 1806 and 1809). Apart from the ARD 1809 sample which shows important age discrepancy linked with analytic limitations, all the other T3 samples follow the Multi-Center approach (slower bleaching kinetics for the Al centers^47^). Al ages show deviations ranging from 100 to 200 ka compared to the Ti-Li ages for ARD 1806, 1809, 1805 and 1808. Such deviation is however less important for ARD 1801 and 1802 samples which yield similar Al and Ti-Li ages within the range of 1 sigma SD. The high bleaching rates affecting all the T3 samples leads to probable age overestimation of the Al centers^27,48^, which makes them unreliable for dating the T3 level (See Supplementary Information, Dating section) and should be considered as maximum age estimations. Nevertheless, Ti-Li centers indicate an effective bleaching rate and can be considered reliable for dating purpose. Although the ARD 1801 and 1802 Al ages fall within the range of their respective Ti-Li ages at 1 sigma SD, they are not considered either for dating for homogeneity purpose. Most of the Ti-Li ages are within the same range (150-200 ka), except for ARD 1801 which delivers Middle-Pleistocene ages (around 500 ka).

To date the T2 level, two samples were taken directly inside the meander cutoff, in a 4-m-deep natural trench in the alluvial sands above the pebble surface (ARD 1803 and ARD 1804). The third sample was taken 10 km upstream, inside the cobble level (ARD 1807). Sediments from this level do not appear to be subject to optical bleaching, so we have no reason to suspect that the ages we obtained were overestimates (Supplementary Table S5). Some of the samples yield slightly older ages for Ti-Li centers (ARD 1803 and 1807). While such configuration is uncommon^49^, the young ages and the high sensibility of Ti-Li centers (low measured values) may lead to potential Ti-Li age overestimation. Nevertheless, all ages remain close within a range comprised within a 100-150 ka range regardless of the center. It thereby confirms the suitability of both Al and Ti-Li centers for dating and that they belong to the same alluvial deposit phase.

## Discussion

**Figure 4:**
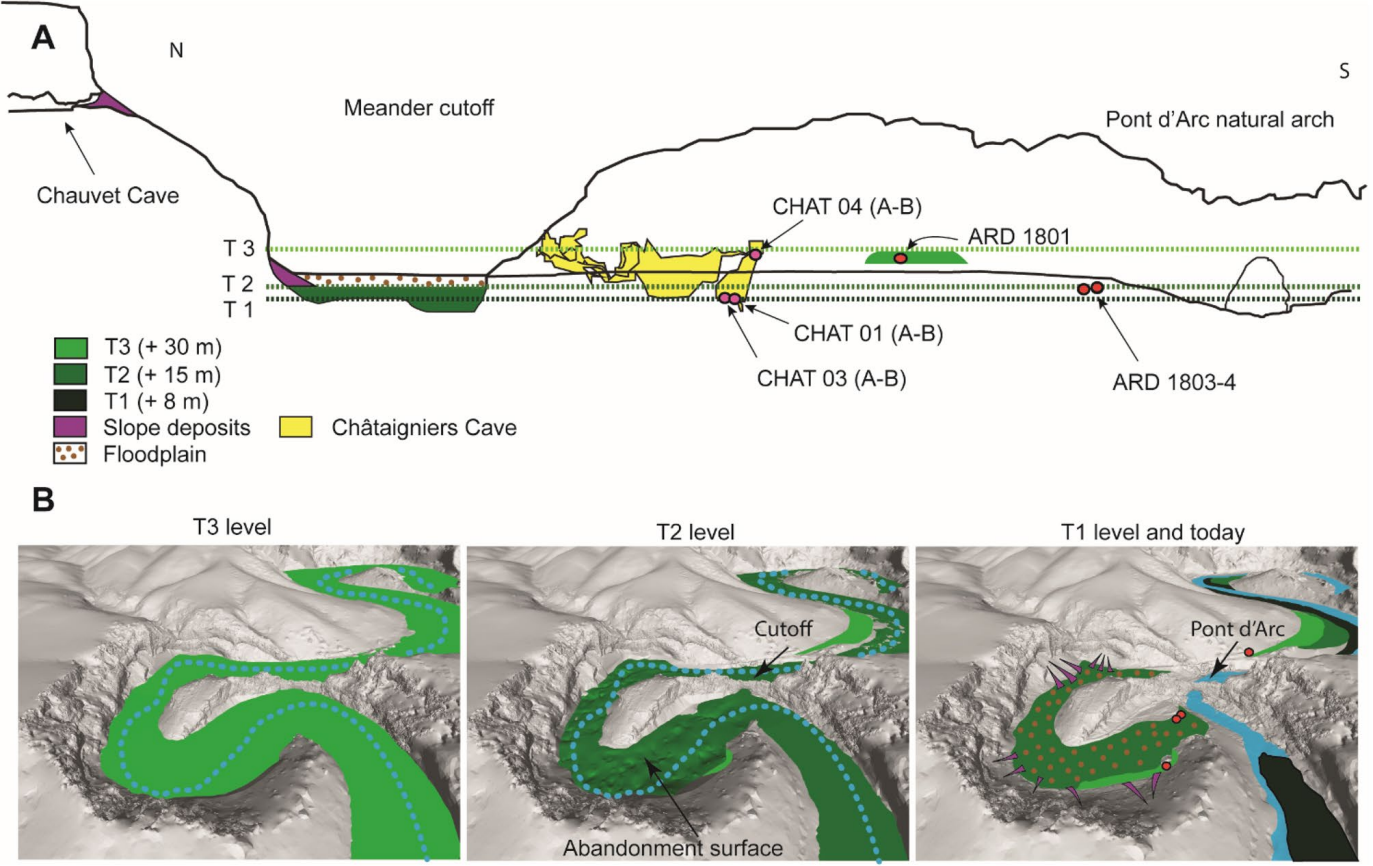
Landscape evolution of the Combe d’Arc meander. (**A**) Cross-section through the Combe d’Arc meander cutoff showing sampling points for the alluvial deposits and speleothems. T1, T2, and T3 correspond to the alluvial deposits at + 8 m, + 15 m, and + 30 m respectively. The diagram shows the altitude of the T1 level in the Combe d’Arc meander cutoff, but this level is not present in this area. 3D reconstruction of the three phases in the meander cutoff process showing theoretical extents of alluvial deposits (green areas).

Combining geomorphological mapping of the sedimentary units in and around the Ardèche River with a geophysical survey and absolute dating of sediment samples enabled to reconstruct the evolution of the Pont d’Arc meander from the Middle Pleistocene to the Holocene (Fig. 4). The following discussion examines the chronological and stratigraphic relationships between external and underground geomorphological features. Features are discussed from oldest to youngest.

At the same altitude than the T3 level, the CHAT-04 speleothem growth stopped at 162.5 ±5 ka and was later covered by laminated clay (Fig. 2). Even if coarse sediments were not discovered in the Châtaigniers cave system, that deposit is linked with a rising of the water level inside the Châtaigniers cave system^20^ and is well above the known Holocene floods of the Ardèche river^40^. We included the date for when the CHAT-04 speleothem stopped growing as a *terminus post-quem* constraint^50^ for the age of T3, as the speleothem could not have continued growing under the T3 water table. The T3 sedimentation phase occurred in all the river system and display large cobbles that are typical of high-energy hydrological conditions. Such processes might have impacted the bleaching rates of the sampled quartz sand^51,52^, but contamination by unbleached quartz grains coming from in situ weathered granite cobbles remains also possible. Most of the Ti-Li ages from the T3 level are comparable with the Optically Stimulated Luminescence (OSL) age (142.5 ±9.2 ka) obtained on fluvial deposits located at 30 m RH in the Ranc Pointu 2 cave^53^, (Fig. 2). Ranc Pointu 2 fluvial deposits also come from the Ardèche river. Considering that we are at the end of the river system (relatively low slope and confluence with the Rhône river), relative heights are comparable before and after the Ardèche Gorges, which allows the stratigraphical attribution of the Ranc Pointu 2 OSL age to the T3 level^22^.

The Middle Pleistocene age of the ARD 1801 sample questions an eventual local reworking of fluvial deposit coming from more ancient levels, or a long-term stability of the T3 level during the middle Pleistocene period. Nevertheless, the addition of the OSL age with the ESR Ti-Li ages (n = 6) produced an estimated Bayesian age of between 136 and 176 ka (95%), with a mean value of 158 ka, that is, during Marine Isotope Stage (MIS) 6 (Supplementary Table S7). In the Combe d’Arc, subsequent erosion has removed most of the cobble layers that formed at the top of T3, but the meander still contains remnants of these deposits (Fig. 4). Their perched position indicates that they were eroded by the river that passed through the old meander. Because the Pont d’Arc natural arch and the T3 alluvial deposit are at almost the same altitude, the cutoff could not have started before the T3 level formed.

**Figure 5:**
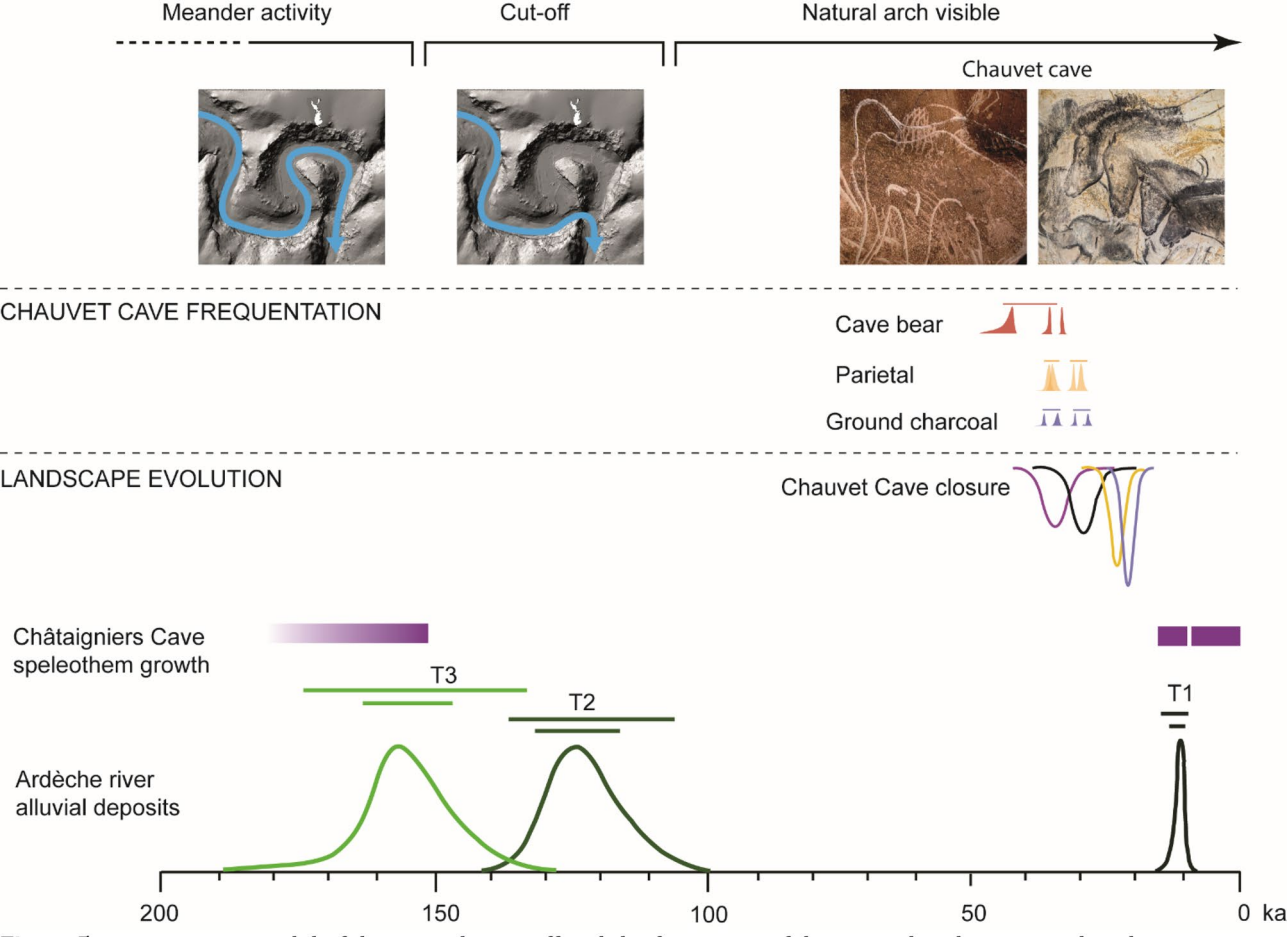
Bayesian age model of the meander cutoff and the formation of the natural arch compared to the presence of humans in the Chauvet Cave. The chronological framework of the cave is based on radiocarbon dating^13^ and ^36^Cl dating of the rockfalls that closed the cave entrance^14^. Photographs of Chauvet Cave artwork: Stéphane Jaillet.

In order to constrain the T2 age, we used the Al and Ti-Li ages of the 1803, 1804 and 1807 ESR samples (n = 6) extracted from sand deposits that correspond to that sedimentation stage. T2 deposits contain few basalt fragments, whereas T1 deposits and the current alluvial plain contain a lot of basaltic material, probably derived from the Coirons lava (dated to between 8 and 5.5 Ma^54^), and the Bas-Vivarais lava (dated to the late-Pleistocene^43^). All ESR ages from the T2 alluvial formation indicate deposition prior to 91 ±13 ka, which is in good agreement with the dates of the last volcanic phases in the Ardèche catchment, around 80 to 35 ka. The Bayesian age obtained for the +15 m level (T2) ranges between 108 ka and 138 ka (95%), with a mean of 124 ka, which shows that this level formed during MIS 5d or MIS 5e (Supplementary Table S7). Absolute dating of the meander abandonment surface shows that the meander became definitively cutoff between 108 and 138 ka (95%), well before the Upper Palaeolithic frequentations (Fig. 5). Although the natural arch probably started to form before that time, the channel through the arch would have been no more than a few meters wide, a similar size to other cave drains within the Pont d’Arc bedrock^20^. Chemical and mechanical weathering, combined with fluvial and gravitational erosion, continued widening the arch until it reached its current size^14,55,56^. Once the meander had been cut-off completely, there would have been little flow in the former river bed, although it has been submerged by flood events during the Holocene and until 1890^40^. These flood events deposited the low-resistivity (<250 Ωm), clay-rich sediments that form the upper part of the Combe d’Arc sediment sequence^40^ (Fig. 2).

Because the T1 alluvial sediments lie below the level of the meander cutoff (+8 m), and therefore do not occur within it, they must have been deposited after the meander became cutoff. The CHAT-03 speleothem grew on top of the corresponding +8 m deposit in the Châtaigniers Cave. Because these clay deposits are at the same altitude as the T1 alluvial sediments, we feel justified in attributing the U-series ages obtained on the speleothem to these sediments (Fig. 5). The stability and age of the + 8 m clay deposit is confirmed by the CHAT-01 ages which span most of the Holocene period. Then, floods affecting the Combe d’Arc meander did not seem to have consequences on the clay deposition in the Châtaigniers cave. Hence, the T1 level probably formed between 11 and 14 ka, that is, at the end of MIS 2, immediately after the last glacial maximum (Fig. 5 and Supplementary Table S7).

## Conclusion

Modeling geomorphological changes to the entrance to the Ardèche Gorge and dating the meander cutoff enabled to reconstruct the area’s landscape during the Upper Paleolithic. The well-constrained age of between 108 and 138 ka for the final meander cutoff yields evidence of the existence of the Pont d’Arc in the Palaeolithic landscape (Fig. 5). The natural arch was highly visible in the river, at the entrance of the Ardèche Gorges. Considering its location and the Upper-Palaeolithic site’s concentration around the Combe d’Arc, we can state that it was unmissable by the Upper Palaeolithic societies. The arch was an element of the cultural landscape of the Upper Paleolithic societies, in the same way as the former Chauvet cave entrance^14^. It shows the need to take into account the surrounding landscape at larger scale, and its reconstruction to its state during the period in question, when studying an archaeological site. This work shows how an integrated geomorphological study of an archaeological site’s surroundings can contribute to investigations of possible associations between the landscape and past human frequentations. The emphasis on the natural arch as part of the archaeological landscape during the Upper-Palaeolithic provides a solid foundation for discussing the notions of "site complexes" and "sense of place"^1,16,57,58^ for archeological sites. Future research will try to answer these questions.

## Methods

### Topography

We used the results of a series of field surveys to produce topographical maps of the sediment deposits inside the meander and on the Vallon Pont d’Arc plain. Given the size of the meander, we used terrestrial Light Detection and Ranging (LiDAR)^20^ to map these morphodynamic features across the Combe d’Arc. The resulting digital elevation model (DEM), obtained after manual and automatic vegetation filtering using CloudCompare software^59^, had a resolution of 5 m. This model was used to map alluvial deposits at 8 m (T1), 15 m (T2), and 30 m (T3) above the current river level. We also mapped the Châtaigniers Cave^20^ so we could sample speleothems in positions exactly 8 m and 30 m above the current river level (Fig. 2).

### Geophysical tomography

Because existing data on the sediments in the Combe d’Arc^40^ were limited to the uppermost 3 m and did not cover the entire meander, we conducted an electrical resistivity tomography survey of the whole site in 2017-2018. For this we used an ABEM multi-electrode system to obtain 44 electrical resistivity profiles involving a total of 2,111 electrode positions, spaced at 5-m intervals (Fig. 3). We measured each electrode’s coordinates with a handheld kinematic Global Positioning System, which allowed us to determine positions to within 3 cm. We used parallelized E4D code^60^ to perform a 3D Occam’s inversion of the electrical resistivity data and selected the 10th iteration as the final electrical resistivity model, with an RMS of 3.3 (see Supplementary Fig. S1 for details of the inversion convergence). The results of this survey enabled us to correlate sediments in the Combe d’Arc with alluvial deposits elsewhere in the Ardèche catchment.

### Sediment sampling

We established an absolute timescale for the evolution of the river landscape by dating alluvial deposits and speleothems. Fluvial sediments were sampled for ESR on quartz dating at T2 and T3 levels. The T1 level was excluded from the sampling because it is often covered by historical flood deposits^40^, and the visible parts are usually in the middle of the river and thus prone to remobilization. The T2 level (ARD 1803, 1804 and 1807) was sampled in yellow middle to coarse grain sized sand lenses and layers located in between pebble layers. For the T3 level, we focused on the sand matrix in between pebbles. We avoided sampling next to weathered granite cobbles and could find middle to coarse grain sized sand lenses for ARD 1805, 1806 and 1808, contrary to ARD 1801, 1802 and 1809.

In the Châtaigniers cave system, speleothems trapped or in contact with clay deposits were sampled for U-series dating on a + 8 m RH well laminated clay level (CHAT-02 and CHAT-03) and inside a laminated clay level at + 30 m RH (CHAT-04). The top and bottom parts of the speleothems were sampled for U-series dating in order to obtain insight on the speleothems growth periods. We assume the sampled speleothems grew if they were above the water table. Their growth period and their stratigraphic organization with paleo water table indicators (i.e laminated clay deposits) or a corresponding altitude with sediments observed outside the cave system can provide constraints for discussing the age of fluvial deposits. Speleothems were chosen for their relative height above the river which corresponded to the T1 and T3 alluvial deposits ones. No speleothem corresponding to the T2 height was found inside the cave.

### Dating

ESR analyses of quartz-rich T3 and T2 alluvial sediments, carried out at the Museum National d’Histoire Naturelle in Paris, provided burial ages for these sediment layers^51,61–64^. We used the multiple paramagnetic centers approach to date the sample^47,65–67^. Analytical uncertainties are reported as ±1 σ errors. We derived Al and Ti-Li ages for each sample (Table 2 and Supplementary tables S4 and S5).

**Table 2:** ESR ages and weighted mean ages for the nine sediment samples.

Ref	Sedimentary unit	Lithology	Sample situation	Age
ARD 1801 Al	T3	Quartz sediment	Pebble-rich Orange sand matrix	511 ± 39
ARD 1801 Ti-Li	–	–	–	489 ± 26
ARD 1802 Al	T3	Quartz sediment	Layered orange sand	186 ± 29
ARD 1802 Ti-Li	–	–	–	185 ± 27
ARD 1805 Al	T3	Quartz sediment	Layered brown sand	304 ± 23
ARD 1805 Ti-Li	–	–	–	202 ± 19
ARD 1806 Al	T3	Quartz sediment	Pebble-rich yellow sand matrix	350 ± 50
ARD 1806 Ti-Li	–	–	–	231 ± 31
ARD 1808 Al	T3	Quartz sediment	Layered orange sand	389 ± 96
ARD 1808 Ti-Li	–	–	–	179 ± 44
*ARD 1809 Al*	*T3*	*Quartz sediment*	*Pebble-rich orange sand matrix*	*215* ± *19*
*ARD 1809 Ti-Li*	–	–	–	*372* ± *28*
ARD 1803 Al	T2	Quartz sediment	Layered yellow sand	106 ± 5
ARD 1803 Ti-Li	–	–	–	159 ± 11
ARD 1804 Al	T2	Quartz sediment	Layered yellow sand	121 ± 13
ARD 1804 Ti-Li	–	–	–	122 ± 15
ARD 1807 Al	T2	Quartz sediment	Yellow Sand lens	91 ± 13
ARD 1807 Ti-Li	–	–	–	131 ± 4

The final column shows the mean age for each group of samples. All ages are given in ka with a 1 σ SD. The ARD 1809 sample (italics) is not considered suitable for dating because of analytical limitation.

Three speleothem samples (CHAT-01-2018, CHAT-03-2018 and CHAT-04-2018) were sent to the Laboratoire des Sciences du Climat et de l’Environnement in Paris, for U-series dating. Analyses were performed on a Multicollector Inductively Coupled Plasma Mass Spectrometer according to a standardized protocol^68^. Analytical uncertainties are reported as ±2 σ errors (Table 1). The dates obtained for CHAT-03-2018 allowed us to constrain the age of the +8 m (T1) river level. We then used Chronomodel software to convert these ESR Al and Ti-Li values along with U-series dates into a Bayesian model with ±2 σ uncertainties^69–71^. We entered prior assumptions, for example, the principles of river incision (level T3 is older than T2, which is older than T1) and of stratigraphic superposition (of speleothems and underground clay deposits), into the relative model in order to test the dating results and obtain final Bayesian ages for each river level (Supplementary Table S7).

## Supplementary Information

Supplementary Files.
