# Author Correction: Dating the landscape evolution around the Chauvet-Pont d’Arc cave

**DOI:** 10.1038/s41598-021-95754-5

**Published:** 2021-08-10

**Authors:** Kim Genuite, Jean‑Jacques Delannoy, Jean‑Jacques Bahain, Marceau Gresse, Stéphane Jaillet, Anne Philippe, Edwige Pons‑Branchu, André Revil, Pierre Voinchet

**Affiliations:** 1 Laboratoire Environnements Dynamiques et Territoires de Montagne, UMR 5204, CNRS, Savoie Mont Blanc University, Campus scientifique, Bat. Pôle Montagne, 73376 Le Bourget‑du‑Lac cedex, France; 2 Histoire Naturelle de L’Homme Préhistorique, UMR 7194, CNRS, MNHN, UPVD, 1, rue René Panhard, 75013 Paris, France; 3 Earthquake Research Institute, University of Tokyo, Tokyo, 158‑8557 Japan; 4 Laboratoire de Mathématiques Jean Leray, Nantes University, 2, rue de la Houssinière, BP 92208, 44322 Nantes, France; 5 Laboratoire Des Sciences du Climat Et de L’Environnement, UMR 8515 CEA, CNRS, UVSQ, Orme des Merisiers, Bat 714, Chemin de Saint Aubin – RD 128, 91191 Gif sur Yvette cedex, France

Correction to: *Scientific Reports* https://doi.org/10.1038/s41598-021-88240-5, published online 26 April 2021

The original version of this Article contained an error in the spelling of the author Anne Philippe, which was incorrectly given as Anne Phillippe. The original Article and accompanying Supplementary Information file have been corrected.

