# Supplementary material for: Dating the landscape evolution around the Chauvet-Pont d’Arc cave
Source: Sci Rep. 2021 Apr 26;11:8944. doi: 10.1038/s41598-021-88240-5 (PMC8076206; doi:10.1038/s41598-021-88240-5)
Supplement: Supplementary file 1 — Supplementary Files. [file 41598_2021_88240_MOESM1_ESM.docx]

**Supplementary Information**

**Dating the landscape evolution around the Chauvet-Pont d’Arc cave**

# Kim Genuite^1,*^, Jean-Jacques Delannoy^1,+^, Jean-Jacques Bahain^2,+^, Marceau Gresse^3,+^, Stéphane Jaillet^1,+^, Anne Philippe^4,+^, Edwige Pons-Branchu^5,+^, André Revil^1,+^, and Pierre Voinchet^2,+^

^1^Laboratoire Environnements et Dynamiques des Territoires de Montagnes, UMR 5204, CNRS, Savoie Mont Blanc University, Campus scientifique, Le Bourget-du-Lac cedex 73376, France.

^2^Histoire Naturelle de l’Homme Préhistorique, UMR 7194, CNRS, MNHN, Sorbonne Universités, Institut de Paléontologie Humaine, 1 rue René Panhard, Paris, 75013, France.

^3^Earthquake Research Institute, University of Tokyo, Tokyo, 158-8557, Japan.

^4^Nantes University, Laboratoire de Mathématiques Jean Leray, 2, rue de la Houssinière, BP 92208, Nantes, 44322, France.

^5^Laboratoire des Sciences du Climat et de l’Environnement, UMR 8212 CEA, CNRS, UVSQ, Orme des Merisiers, Bat 714, Chemin de Saint Aubin – RD 128, Gif sur Yvette cedex, 91191, France.

This PDF file includes:

- Supplementary text

- Supplementary references

- Figures S1 to S5

- Tables S1 to S7

# Supplementary Information

**SI Electrical resistivity inversion modelling**

Measurements were conducted with an acquisition time of 0.3 s, temporized at 0.5 s. The injected current ranged from 1 mA to 200 mA, depending on the resistance of the subsoil. The sandy-clay substratum gave very good resistance contacts (< 10 kΩ). The raw dataset contained 12,528 apparent resistivity values, each of which was obtained by stacking two individual measurements. Measurement errors > 1% were automatically eliminated during acquisition. The remaining data were filtered using a standard deviation threshold of 10% in addition to a visual inspection of the pseudosections. We retained 11,817 apparent resistivity values for the inversion.

Using the TetGen algorithm[1] to construct the computational domain for the Combe d’Arc meander allowed us to produce a 5-m resolution digital elevation model of the gorge’s surface topography. We used a finer mesh around the electrodes in order to obtain more detailed profiles in these areas. The resulting computational domain consisted of 157,017 nodes and 707,953 unstructured tetrahedral elements covering an area of 0.74 km² (Fig. S1).

We used parallelized E4D code[2] to perform an Occam’s inversion on the electrical resistivity data. To avoid overfitting the data, we carefully analyzed the inversion results at each iteration. A satisfactory resistivity model, with an RMS error of 3.3, was selected after 10 iterations (Table S1).

We interpreted resistivity values of 250 Ωm as indicating the upper limit of the T2 alluvial deposit. We used this isovalue to map the extent of this unit within the meander. The mean distribution was estimated from manually digitized polylines drawn on the 2D inversion profiles. Each polyline was then plotted in a 3D environment using 3DReshaper software. Analysis of the point cloud extracted from the polylines, conducted using CloudCompare, produced a gaussian distribution, which suggests that the deposit’s topography is relatively uniform. Hence, we used the ±1 σ distribution value to determine the altitude of the top of the T2 deposit.

**SI Dating**

**Electron spin resonance dating method.**

In line with the protocol described by[3], we sieved each sample in order to extract the 100-200 µm fraction [4], [5], [6], [7] and then removed any remaining carbonates in this fraction by adding hydrochloric acid and washing the residue in water and acetone. After drying at 40°C, we immersed the samples in hydrofluoric acid (40%) for 2 h before removing heavy and magnetic minerals using heavy liquids (sodium polytungstate) and magnetic separation, respectively. We then divided the purified quartz grains into 11 equal parts called aliquots.

We determined the bleaching rate (δbl %) for each sample by exposing one of the 11 aliquots to light for 1600 h in a Dr Honhle© SOL2 solar simulator (light intensity between 3.2 and 3.4^.^10^5^ Lux) in order to measure the unbleachable portion of the ESR-Al signal[3], and then comparing the ESR intensity of this bleached (Ibl) aliquot with the natural ESR intensity (Inat) measured on one of the remaining aliquots in order to = (δbl (%) = (Inat-Ibl)/Inat) x 100). ESR intensity linked to the unbleachable component was then corrected prior to age calculation.

The other nine aliquots were exposed to different doses of gamma radiation in order to determine the equivalent dose (additive method), that is, the amount of radiation received by the sample since it was buried. The samples were irradiated by a γ ^137^Cs source (CENIEH, Burgos, Spain) with a dose rate of about 6 Gy/min to give nine additive doses of 150, 300, 600, 1,200, 2,400, 4,000, 6,000, 8,000, and 12,000 Gy. Analyses were conducted between 105 and 112 K using a Brucker EMXplus Electron Spin Resonance spectrometer. We analyzed each of the eleven aliquots of each sample nine times (on three different days and at three different angles), adopting a multiple-center approach (measuring both the Al and Ti-Li centers)[8], [9], [10]. However, for operational reasons relating to the signal’s high sensitivity[10] and the limitations of the MNHN ESR spectrometer, we did not calculate ages from the Ti-H signal. ESR intensities were calculated using the mean value of the three signal intensities and were normalized for aliquot weight. Aliquots for which measurements differed by more than 10% were excluded from the paleo-dose calculations. We performed a baseline correction using WINEPR software to extract the ESR intensities. For the Al signals, measurements were performed between the top of the first peak (g=2.018) and the bottom of the sixteenth peak (g=2.002). In line with[8] recommendations, we evaluated the ESR intensity of the Ti-Li signal by measuring the peak-to-baseline amplitude at around g = 1.913-1.915 (Fig. S4). The following ESR acquisition parameters were selected: microwave power = 5 mW, point resolution = 1,024, sweep width = 20 mT, modulation frequency = 100 kHz, modulation amplitude = 0.1 mT, conversion time = 40 ms, time constant = 20 ms, and 1 scan.

We calculated ages for the Al and Ti-Li centers for each sample by plotting dose response curves from the mean values of the spectrometer runs (three days of measurement) (Table S3).

Growth curves were obtained by plotting ESR intensities against the dose received by each aliquot. Equivalent doses values (De) were obtained by back extrapolation to Y=0. We determined equivalent doses (De) by using Microcal OriginPro 8 software and a Levenberg-Marquardt algorithm to perform chi-square minimization. Data were weighted using the inverse of the squared ESR intensity (1/I^2^) for each sample. Al dose response curves (DRC) were fitted by applying an exponential + linear function (EXP+LIN) for all the samples[11], (Fig. S3).

Ti DRC are commonly described by SSE applied on the low irradiated intensity points [12], [13] and Ti2 function that allow the description of its saturation or decrease for high doses[11], [14], [15]. Some of the Ti-Li DRC could be correctly described by SSE, while others describe a SSE function at the beginning, and follow a linear term after the main kink, which shows that traps are continuously generated during the irradiation. Such observations could be related with the high external radiative contribution delivered by the Ardèche river sediments, and their relatively young age. SSE fitting was attempted on all samples but eventually delivered inconsistent results, regarding their goodness of fit, the age (important age overestimation regarding both the Al ages and the general morphodynamical context with U-series), and the general aspect of the growth curves (Fig. S3). Considering that the DRC fitting function choice should be the one that follows the least deviation with the dataset[16], [17], we use the EXP+LIN function on some of the samples as it provides a better description of the Ti-Li DRCs shape (r^2^). SSE fitting was then applied to describe Ti-Li DRC on ARD 1801, 1805, 1806 and 1809 samples, and the EXP+LIN function was preferred for the other ones.

Dose rates (Da) were obtained by combing gamma radiation measurements made in situ (in the field) with alpha and beta measurements made in the laboratory, and then adding the cosmic ray contribution. Gamma dose rates were measured using a Digidart gamma spectrometer (Ortec©) combined with the threshold approach[18]. External alpha and beta contributions were calculated from the sediment’s radioelement contents (U, Th, and K), determined in the laboratory using a high-resolution, low-background gamma-spectrometer[19]. Age calculations were performed using[20] dose-rate conversion factors. We assumed a k-value of 0.15 ±0.1[4], [21]. Alpha and beta attenuations were taken from[22], [23]. Water attenuation formulae were taken from[24] and a cosmic dose rate was calculated using the equations drawn up by[25] (Tables S3 and S4). The internal dose rate was considered to be negligible because of the very low radionuclide contents of quartz grains[26], [27].

Given the area’s Mediterranean climate (long dry periods punctuated by intense rain events), sediment water contents are likely to have fluctuated widely[7], [28], so we assumed a water content of 15% for all of our samples (Tables S3). This assumption was also influenced by possible variations in sediment water content between glacial and interglacial periods. Such variations have been demonstrated in the neighboring Massif Central[29].

The uncertainty in individual ESR ages results from a ±1 σ propagation of the equivalent dose calculation, given the uncertainties resulting from the assumed water content, depth estimates, in-situ gamma dose rates, beta dose rate attenuation, and cosmic dose rates.

**ESR results**

For the T3 level, the ARD 1801 sample possesses high bleaching rates (75-77 %). Both Al and Ti-Li ages show outlier position compared to the other samples (more than 100 ka older). Al and Ti-Li ages respect the MC approach and are close to each other at ±1 σ (511 ±39 ka and 489 ±26 ka respectively).

The ARD 1802 possesses bleaching rates below 70 %. The sample shows good fitting for Al age (r^2^ > 0.98) but less good one for Ti-Li ages (r^2^ between 0.97 and 0.98) and show deviations within the range of ±1 σ between the ages provided by the two centers (186 ±29 ka and 185 ±27 ka). While the Al fitting remains consistent regarding the DRC shape (Fig. S3), the poor fitting result is more visible for the Ti-Li center. Such discrepancy may be caused to the high variability of the upper part of the DRC after the main kink.

The ARD 1805 sample also shows bleaching rates below 70 %. Both Al and Ti-Li ages show moderate to excellent goodness of fit (r^2^ > 0.98 and 0.99), and propose ages that are consistent with the MC approach (304 ±23 ka and 202 ±19 ka). The important deviation between Al and Ti-Li ages (100 ka) is probably related to Al age overestimation linked with the high bleaching rates.

The ARD 1806 sample is affected by high bleaching rates (> 87 %). Al and Ti-Li ages are consistent with the MC approach (350 ±50 ka and 231 ±31 ka) and show moderate to excellent goodness of fit (r^2^ > 0.98 and > 0.99 respectively). The high bleaching rates also lead to a probable age overestimation of the Al centers, hence a deviation of more than 100 ka between the two ages.

The ARD 1808 sample is affected by moderate to high bleaching rates (65 %). Al and Ti-Li ages are consistent with the MC approach and yield excellent goodness of fit for both centers (r^2^ > 0.99 and 0.98). They also show a probable age overestimation for the Al centers with more than 200 ka of difference (389 ±96 ka and 179 ±44 ka).

The ARD 1809 sample shows high bleaching rates (81 %). Al and Ti-Li ages do not follow the MC approach and show age deviations superior to 150 ka. This may be explained by the unreliability of the spectrometer during the ARD 1809 run (low quantity of analysed quartz material). We then rule out the sample prior to any interpretation. In line with the MC approach[8], the general high bleaching rates affecting the samples suggests that the signal of the Al center has been incompletely reset during the sediment transportation. Consequently, the Ti-Li provides the best estimation for the burial age of the T3 samples[30].

For the T2 level, all samples show normal to moderate bleaching rates. They do not all respect the MC approach. This can be related to the relatively young ages (around 100-150 ka for all the T2 ages) that leads to low Ti-Li signal, making it difficult to measure, which leads to slight overestimation when compared with the Al ages.

This is the case for the ARD 1803 sample: Al and Ti-Li ages show moderate deviation (respectively 106 ±5 ka and 159 ±11 ka) and good to excellent goodness of fit (r^2^ > 0.98 and 0.99). The older Ti-Li age is however consistent with the Ti-Li ages obtained on the other T2 samples, which questions the reliability of the Al age which may be slightly underestimated. The ARD 1804 shows Al and Ti-Li ages of 121 ±13 and 122 ±15 ka. They are consistent with the MC approach within the range of 1 sigma SD. Finally, the ARD 1807 sample shows Al and Ti ages (91 ±13 ka and 131 ±4 ka) that do not respect the MC approach despite showing excellent goodness of fit (r^2^ > 0.99). Nevertheless, those remain close at ±2 σ. Despite occasional deviation in the MC approach, the global coherence of the Al and Ti-Li ages for the T2 level leads to consider both centers for dating.

**U-series dating**

We used a diamond micro saw to sample the top and base of each stalagmite. Samples of between 150 and 300 mg were used for U-Th analysis. After adding a triple ^229^Th, ^233^U, ^236^U spike in a Teflon beaker, samples were dissolved with dilute HCl. U-Th separation and purification were performed after coprecipitation with Fe(OH)_3_ in 0.6 ml columns filled with U-TEVA® and pre-filter resins. U and Th isotope analyses were carried out at the Laboratoire des Sciences du Climat et de l’Environnement (France) on a Thermo Scientific^TM^ Neptune^Plus^ multi-collector inductively coupled plasma source mass spectrometer fitted with a desolvating sample introduction system (Aridus II) and a jet pump interface. For mass fractionation correction, we used an exponential mass fractionation law (normalized to natural ^238^U/^235^U isotopic ratio) and standard/sample bracketing. A more detailed description of the analytical procedure (chemistry and MC-ICPMS analysis) can be found in[31]. After correcting for peak tailing, hydrate interference, and chemical blanks, we used iterative age estimation to calculate ^230^Th/^234^U ages (Table 1) from the measured atomic ratios, applying the ^230^Th, ^234^U and ^238^U decay constants provided by[32] and[33] (Tables 1 and S6).

**Bayesian Statistical Modelling**

In order to test our hypothesis that the Pont d’Arc natural arch would have been visible to the creators of the Chauvet Cave’s rock art, we divided the U-series, ESR, and OSL dates into four phases, which occurred in the following order: CHAT-04 speleothem growth > T3 level > T2 level > T1 level. We used this succession as our *a priori* chronological model. We assumed that the ages obtained for each level correspond to a single geomorphological object, so we combined them to obtain one age for each level[34].

Bayesian age modelling was performed using Chronomodel 2.0.18 software[35], [36] and assuming a gaussian distribution for the U-series, OSL, and ESR ages. The relative phase model was based on the karst-river stratigraphical relationships[37] between the sediments in the Châtaigniers Cave and the alluvial deposits (Fig. 2). We followed the stratigraphy shown in Table S7 when entering data into Chronomodel.

We used Chronomodel’s age modelling function (called TL/OSL), to enter the dates with their ±1 σ errors. Applying a Metropolis-Hastings algorithm[38] gave a prior distribution for each date. This method is recommended for dates obtained using methods other than radiocarbon dating[36]. We used three Monte-Carlo Markov Chains (MCMC) for the calculations, performing 1,000 iterations for each one. Brun-in phases: 1,000 iterations, batches: 1000 iterations, acquisitions: 10,000 iterations (Table S7).

**
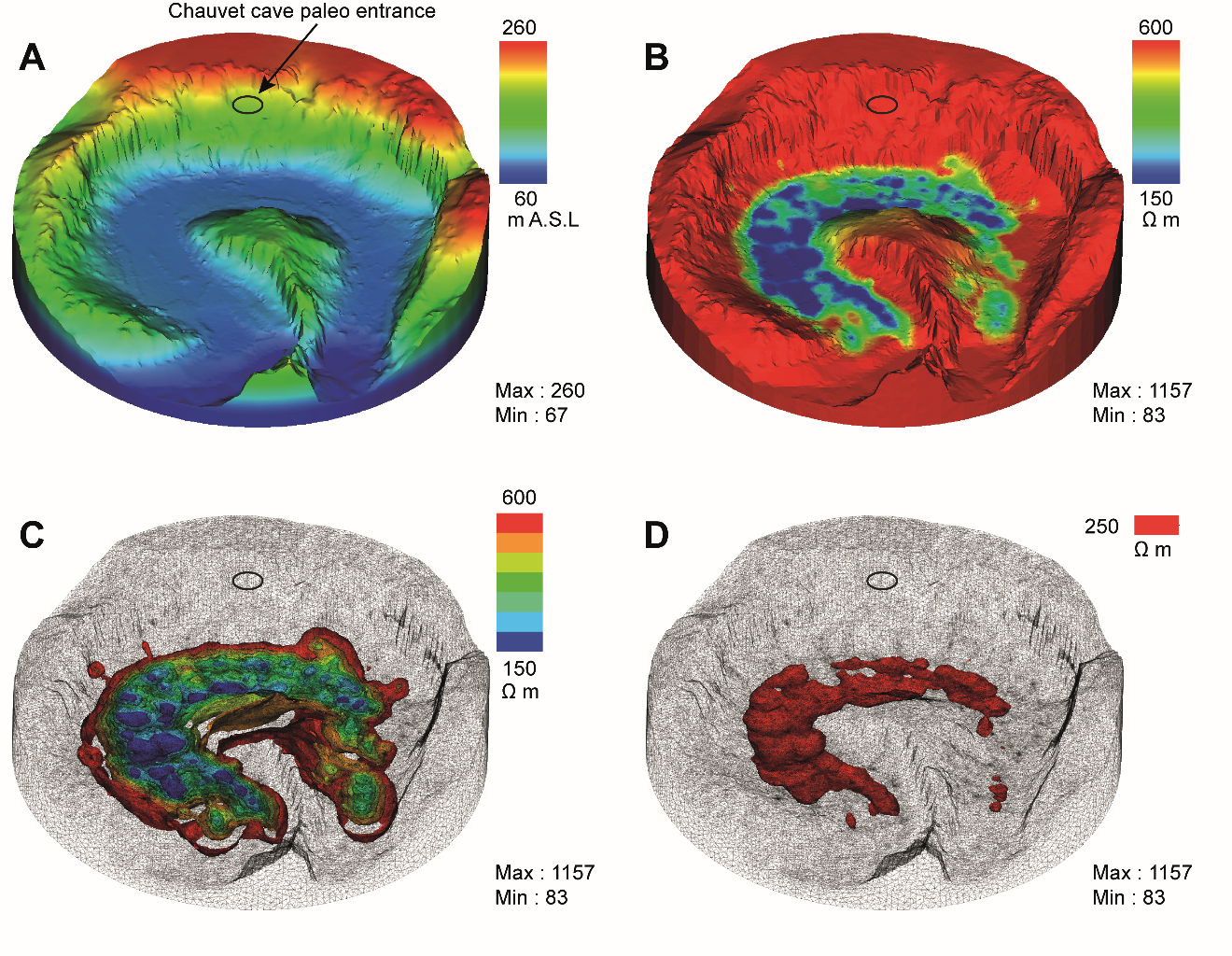
**

**Supplementary Figure S1.** 3D electrical resistivity models for the Combe d’Arc meander. A. Topography of the mesh domain used for the 3D electrical resistivity inverse modelling inside the Combe d’Arc meander. The mesh, which is denser close to the electrodes, contains 707,953 tetrahedral elements. B. Surface resistivity. C. Plot of resistivity iso-values. D. Plot of 250 Ωm resistivity isovalues. Convergence occurred very quickly, after just a few iterations, which shows that the fieldwork data was coherent across the entire meander.


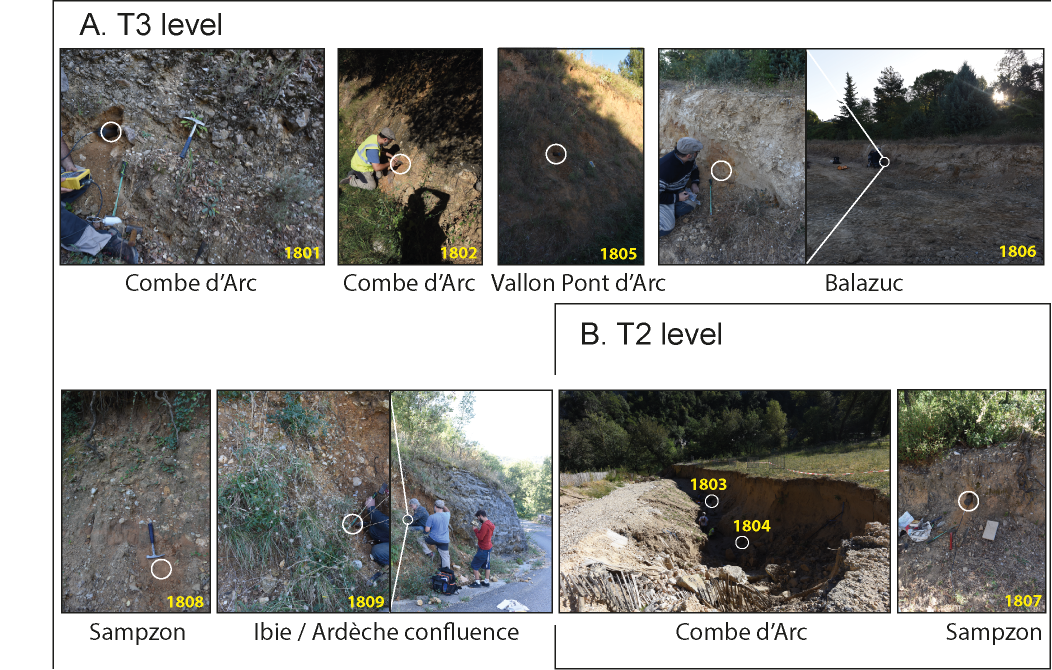


**Supplementary Figure S2.** Photographs of the ESR sampling locations. Most ESR samples from the T3 (A) and T2 (B) levels were taken on the Vallon-Pont-d’Arc plain, in road cuttings. Photographs from: Stéphane Jaillet.

**
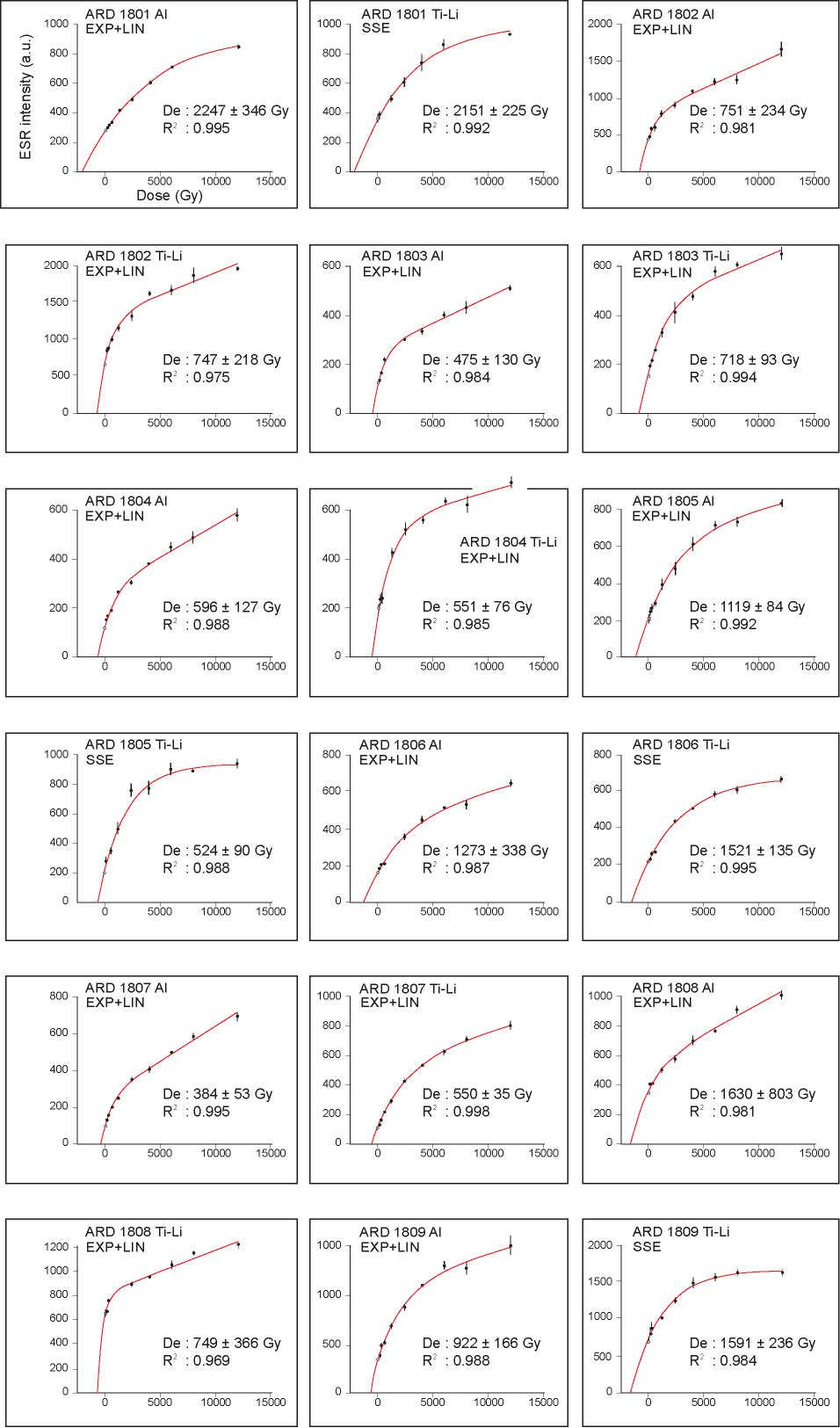
**

**Supplementary Figure S3.** ESR signal dose response curves for each sample. De is given at 2 sigma SD.


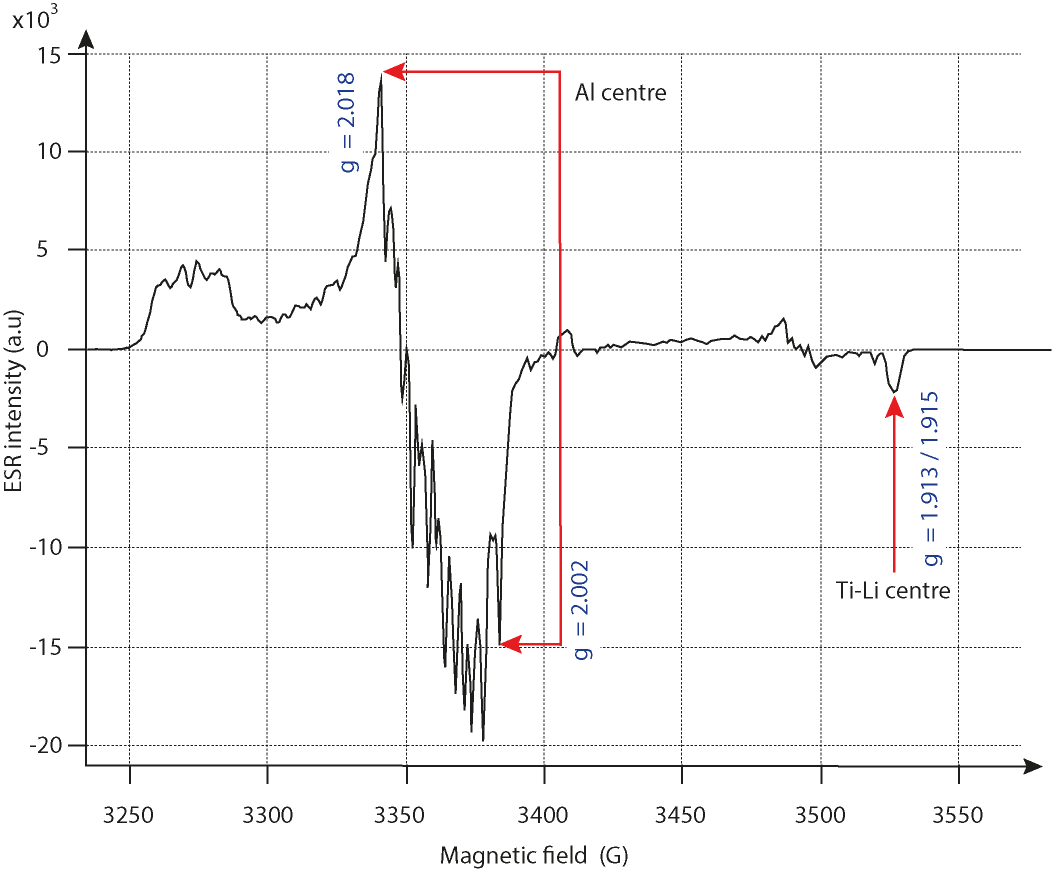


**Supplementary Figure S4.** Example of ESR spectra obtained on a gamma-irradiated ARD 1805 aliquot. Red arrows highlight the chosen measurement options for Al and Ti-Li centers.


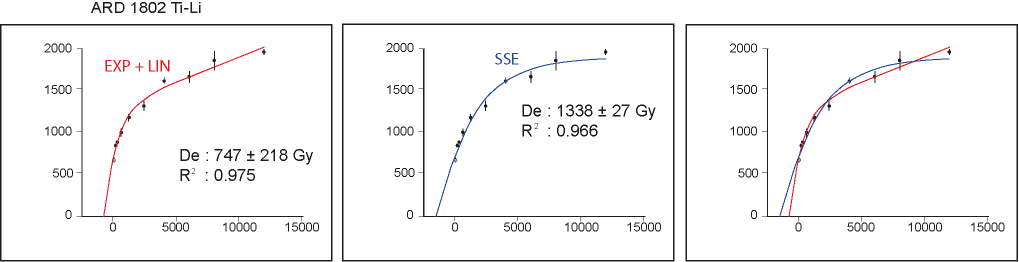


**Supplementary Figure S5.** Example of fitting difference between the EXP+LIN and the SSE function on ARD 1802 Ti-Li DRC. The EXP-LIN function is preferred to the SSE function in some cases as it better describes the Ti-Li DRC morphology and delivers better goodness of fit values.

**Supplementary Table S1.** 3D inversion of the ERT model.

| **Phi_dat** | **Phi_mod** | **Phi_mod/beta** | **Phi_tot** |
| --- | --- | --- | --- |
| 1.18E+05 | 86,384 | 1,382.1 | 2,04E+05 |
|  |  |  |  |
| **Block** | **Nb. Of Constraints** | **% of total error** | **Error per constraint** |
| 1 | 239,8891 | 95,117 | 1.18E-02 |
| 2 | 246,450 | 4,8831 | 5.89E-03 |
|  |  |  |  |
| **Chi2** | **Mean error** | **RMS error** |  |
| 10,9 | -0.4804 | 3.302 |  |

Convergence occurred very quickly, after just a few iterations, which shows that the fieldwork data was coherent across the entire meander.

**Supplementary Table S2.** Coordinates of the ESR sampling points for the +30 m (T3) and +15 m (T2) alluviums deposited by the Ardèche River.

| **Ref.** | **Strati.** | **Lat.** | **Long.** | **Altitude (m above sea level)** | **Depth (cm)** |
| --- | --- | --- | --- | --- | --- |
| ARD 1801 | T3 | 44.383361 | 4.412542 | 100 | 400 |
| ARD 1802 | T3 | 44.378947 | 4.418604 | 104 | 400 |
| ARD 1805 | T3 | 44.401531 | 4.386502 | 108 | 100 |
| ARD 1806 | T3 | 44.523235 | 4.403885 | 160 | 400 |
| ARD 1808 | T3 | 44.419877 | 4.352217 | 124 | 150 |
| ARD 1809 | T3 | 44.392737 | 4.403177 | 108 | 200 |
| ARD 1803 | T2 | 44.382523 | 4.415352 | 85 | 400 |
| ARD 1804 | T2 | 44.382523 | 4.415352 | 85 | 400 |
| ARD 1807 | T2 | 44.423838 | 4.348955 | 105 | 400 |

**Supplementary Table S3.** Radioelement contributions to annual doses for the alluvial deposit samples.

| **Ref.** | **U (dpm)** | **Th (dpm)** | **K (%)** | **Water (%)** |
| --- | --- | --- | --- | --- |
| ARD 1801 | 3.01 ±0.08 | 3.98 ±0.04 | 3.34 ±0.02 | 15 |
| ARD 1802 | 1.36 ±0.07 | 1.81 ±0.03 | 3.85 ±0.02 | 15 |
| ARD 1805 | 2.39 ±0.09 | 3.45 ±0.05 | 2.49 ±0.02 | 15 |
| ARD 1806 | 1.42 ±0.07 | 1.65 ±0.04 | 3.87 ±0.03 | 15 |
| ARD 1808 | 1.58 ±0.08 | 2.19 ±0.04 | 3.80 ±0.03 | 15 |
| ARD 1809 | 1.73 ±0.08 | 2.10 ±0.04 | 3.96 ±0.03 | 15 |
| ARD 1803 | 1.93 ±0.08 | 2.24 ±0.04 | 3.78 ±0.03 | 15 |
| ARD 1804 | 2.87 ±0.09 | 2.90 ±0.04 | 3.79 ±0.02 | 15 |
| ARD 1807 | 2.12 ±0.09 | 2.39 ±0.04 | 3.26 ±0.03 | 15 |

**Supplementary Table S4.** Radiative contributions to annual dose rates for the alluvial deposit samples.

| **Ref.** | **Dα (µGy/a)** | **Dβ (µGy/a)** | **Dɣ (µGy/a)** | **Cosmic (µGy/a)** |
| --- | --- | --- | --- | --- |
| ARD 1801 | 115 ±2 | 2.786 ±26 | 1.397 ±70 | 103 ±5 |
| ARD 1802 | 52 ±2 | 2.703 ±26 | 1.190 ±60 | 103 ±5 |
| ARD 1805 | 96 ±2 | 2.134 ±29 | 1.290 ±65 | 166 ±8 |
| ARD 1806 | 50 ±2 | 2.708 ±28 | 760 ±38 | 180 ±9 |
| ARD 1808 | 62 ±2 | 2.732 ±30 | 1.247 ±62 | 152 ±8 |
| ARD 1809 | 62 ±2 | 2.839 ±29 | 1.239 ±62 | 140 ±7 |
| ARD 1803 | 68 ±2 | 2.770 ±29 | 1.526 ±76 | 116 ±6 |
| ARD 1804 | 94 ±2 | 2.957 ±30 | 1.775 ±89 | 103 ±5 |
| ARD 1807 | 73 ±2 | 2.488 ±31 | 1.550 ±78 | 103 ±5 |

**Supplementary Table S5.** ESR paramagnetic centers used for the equivalent dose calculations.

| **Ref.(*)** | **δbl (%)** | **r^2^** | **Da (µGy/a)** | **De (Gy)** |
| --- | --- | --- | --- | --- |
| ARD1801 Al | 77 | 0.995 | 4,400 ±77 | 1247 ±173 |
| ARD1801 Ti-Li | 100 | 0.992 |  | 1754 ±113 |
|  |  |  |  |  |
| ARD1802 Al | 68 | 0.981 | 4,047 ±68 | 751 ± 117 |
| ARD1802 Ti-Li | 100 | 0.975 |  | 747 ±109 |
|  |  |  |  |  |
| ARD1805 Al | 69 | 0.992 | 3,663±75 | 1119 ±42 |
| ARD1805 Ti-Li | 100 | 0.988 |  | 524 ±45 |
|  |  |  |  |  |
| ARD1806 Al | 87 | 0.987 | 3,699 ±52 | 1273 ±169 |
| ARD1806 Ti-Li | 100 | 0.995 |  | 1521 ±68 |
|  |  |  |  |  |
| ARD1808 Al | 65 | 0.981 | 4,193 ±72 | 1630 ±402 |
| ARD1808 Ti-Li | 100 | 0.969 |  | 749 ±183 |
|  |  |  |  |  |
| ARD1809 Al | 81 | 0.988 | 4,281 ±72 | 922 ±83 |
| ARD1809 Ti-Li | 100 | 0.984 |  | 1591 ±118 |
|  |  |  |  |  |
| ARD1803 Al | 52 | 0.984 | 4,480 ±84 | 475 ±65 |
| ARD1803 Ti-Li | 100 | 0.994 |  | 718 ±47 |
|  |  |  |  |  |
| ARD1804 Al | 50 | 0.988 | 4,929 ±97 | 596 ±64 |
| ARD1804 Ti-Li | 100 | 0.985 |  | 551 ±38 |
|  |  |  |  |  |
| ARD1807 Al | 46 | 0.995 | 4,214 ±87 | 384 ±27 |
| ARD1807 Ti-Li | 100 | 0.995 |  | 550 ±18 |

**Supplementary Table S6.** U-series results for the speleothem samples, showing U and Th contents, isotope ratios, and ages.

| **Ref.** | **Lat.** | **Long.** | **Alt.** | **[^238^U] ppm** | **[^232^Th] ppt** | **δ^234^UM** | **(^230^Th/^238^U)** | **(^230^Th/^232^Th)** | **Age** |
| --- | --- | --- | --- | --- | --- | --- | --- | --- | --- |
| CHAT-04A | 44,384679 | 4,415292 | 102.5 | 0.193 ±0.002 | 0.11 ±0.002 | 36.8 ±1.68 | 1.012 ±0.0033 | 5660.17 ±18.21 | 364.24 ±15.5 |
| CHAT-04B | 44,384679 | 4,415292 | 102.8 | 0.255 ±0.002 | 0.08 ±0.002 | 41.32 ±0.9 | 0.8123 ±0.0103 | 8407.01 ±107.08 | 162.46 ±5.06 |
| CHAT-03A | 44,384821 | 4,415152 | 80.0 | 0.197 ±0.002 | 4.55 ±0.04 | 291.42 ±1 | 0.0897 ±0.0013 | 11.92 ±0.17 | 15.66 ±2.91 |
| CHAT-03B | 44,384821 | 4,415152 | 80.2 | 0.192 ±0.002 | 14.11 ±0.11 | 257.4 ±1.0 | 1.1277 ±0.002 | 5.35 ±0.08 | 13.17 ±1.12 |
| CHAT-01A | 44.384821 | 4.415154 | 79.5 | 0.276 ±0.002 | 16.95 ±0.14 | -147.89 ±0.91 | 0.0820 ±0.0004 | 4.04 ±0.02 | 7.25 ±1.95 |
| CHAT-01B | 44.384821 | 4.415154 | 79.6 | 0.117 ±0.001 | 5.65 ±0.05 | -131.65 ±0.73 | 0.0410 ±0.0006 | 2.58 ±0.04 | 2.34 ±1.54 |

U and Th content, isotopic activity ratios and corrected ages for speleothem samples. δ^234^U = ({^234^U/^238^U} _measured_/{^234^U/^238^U} _equilibrium_ - 1) × 1000, with ^234^U/^238^U_equilibrium_ = 54.89×10^-6^ (molar ratio. [29]). Altitudes are shown in meters above sea level (a.s.l).

**Supplementary Table S7.** Bayesian statistical modelling of the T1, T2, and T3 alluvial deposit events at 95% confidence.

| **Modelled phases** | **MAP** | **Mean** | **Std dev** | **Q1** | **Q2** | **Q3** | **Max age range (95 %)** |
| --- | --- | --- | --- | --- | --- | --- | --- |
| CHAT 01 end | 2,3 | 3,1 | 0,9 | 2,4 | 2,8 | 3,5 | [2; 4,9] |
| CHAT 01 start | 7,2 | 7,3 | 1,6 | 6,3 | 7,2 | 8,2 | [3,9; 10,6] |
| T1 (CHAT 03) | 13,5 | 13,8 | 1,1 | 13,1 | 13,6 | 14,3 | [11,9; 16,2] |
| T2 | 126,1 | 124,1 | 7,6 | 119,6 | 124,9 | 129,4 | [108,2; 138,1] |
| T3 | 158,9 | 157,6 | 10,0 | 152,2 | 157,5 | 162,1 | [136,1; 176,3] |
| CHAT 04 end | 163,4 | 166,8 | 12,8 | 161,7 | 164,3 | 167,6 | [152,6; 186,8] |
| CHAT 04 start | 364,3 | 364,0 | 18,3 | 356,3 | 364,2 | 372,0 | [331,9; 397,6] |
| **Modelled ages** |  |  |  |  |  |  |  |
| CHAT 01 end | 2,5 | 2,6 | 0,7 | 2,2 | 2,6 | 3,0 |  |
| CHAT 01 start | 7,1 | 7,2 | 1,0 | 6,5 | 7,2 | 7,9 |  |
| CHAT 03 end | 14,0 | 14,3 | 1,2 | 13,5 | 14,2 | 15,0 |  |
| CHAT 03 start | 13,2 | 13,3 | 0,5 | 12,9 | 13,3 | 13,6 |  |
| ARD 1803 Al | 121,5 | 118,5 | 10,8 | 112,2 | 119,5 | 125,9 |  |
| ARD 1803 Ti-Li | 146,8 | 147,4 | 11,9 | 138,7 | 147,0 | 155,6 |  |
| ARD 1804 Al | 124,5 | 123,1 | 9,0 | 117,6 | 123,6 | 129,2 |  |
| ARD 1804 Ti-Li | 126,1 | 123,5 | 9,2 | 117,8 | 123,9 | 129,6 |  |
| ARD 1807 Al | 109,6 | 106,5 | 13,0 | 98,1 | 107,6 | 116,1 |  |
| ARD 1807 Ti-Li | 129,5 | 129,7 | 3,9 | 127,1 | 129,7 | 132,2 |  |
| OSL Ranc Pointu 2 | 149,7 | 148,7 | 8,5 | 143,2 | 149,0 | 154,5 |  |
| ARD 1801 Ti-Li | 482,5 | 482,8 | 26,4 | 465,4 | 483,0 | 500,5 |  |
| ARD 1802 Ti-Li | 161,2 | 166,7 | 18,4 | 154,8 | 164,5 | 176,9 |  |
| ARD 1805 Ti-Li | 180,9 | 183,8 | 18,7 | 170,0 | 182,7 | 196,2 |  |
| ARD 1806 Ti-Li | 169,5 | 190,4 | 29,4 | 168,0 | 186,2 | 209,2 |  |
| ARD 1808 Ti-Li | 158,7 | 161,6 | 21,5 | 149,3 | 160,0 | 172,3 |  |
| CHAT 04 end | 162,9 | 163,0 | 2,5 | 161,3 | 163,0 | 164,7 |  |
| CHAT 04 start | 363,6 | 364,2 | 7,6 | 359,1 | 364,1 | 369,3 |  |

Ages are expressed in ka BP. MAP: maximum a posteriori probability. Q1,2,3: first to third quartiles of the full age range for the event.

**Supplementary References**

1. Si, H. TetGen, a Delaunay-Based Quality Tetrahedral Mesh Generator. *ACM Trans. Math. Softw.* **41**, 11:1-11:36 (2015).

2. Johnson, T. C., Versteeg, R. J., Ward, A., Day-Lewis, F. D. & Revil, A. Improved hydrogeophysical characterization and monitoring through parallel modeling and inversion of time-domain resistivity andinduced-polarization data. *Geophysics* **75**, WA27–WA41 (2010).

3. Voinchet, P. *et al.* ESR dating of quartz extracted from Quaternary sediments application to fluvial terraces system of northern France. *Quaternaire* **15**, 135–141 (2004).

4. Yokoyama, Y., Falgueres, C. & Quaegebeur, J. P. ESR dating of quartz from quaternary sediments: First attempt. *Nuclear Tracks and Radiation Measurements (1982)* **10**, 921–928 (1985).

5. Aitken, M. J. *Thermoluminescence dating*. (1985).

6. Bell, W. Attenuation factors for the absorbed radiation dose in quartz inclusions for thermoluminescence dating. *Ancient TL* **8**, (1979).

7. Duval, M. *et al.* Assessing the uncertainty on particle size and shape: Implications for ESR and OSL dating of quartz and feldspar grains. *Radiation Measurements* **81**, 116–122 (2015).

8. Toyoda, S., Voinchet, P., Falguères, C., Dolo, J. M. & Laurent, M. Bleaching of ESR signals by the sunlight: a laboratory experiment for establishing the ESR dating of sediments. *Applied Radiation and Isotopes* **52**, 1357–1362 (2000).

9. Tissoux, H. *et al.* Potential use of Ti-center in ESR dating of fluvial sediment. *Quaternary Geochronology* **2**, 367–372 (2007).

10. Duval, M., Sancho, C., Calle, M., Guilarte, V. & Peña-Monné, J. L. On the interest of using the multiple center approach in ESR dating of optically bleached quartz grains: Some examples from the Early Pleistocene terraces of the Alcanadre River (Ebro basin, Spain). *Quaternary Geochronology* **29**, 58–69 (2015).

11. Duval, M. Dose response curve of the ESR signal of the Aluminum center in quartz grains extracted from sediment. *Ancient TL* **30**, 41–50 (2012).

12. Toyoda, S. & Ikeya, M. Thermal stabilities of paramagnetic defect and impurity centers in quartz: Basis for ESR dating of thermal history. *Geochemical Journal* **25**, 437–445 (1991).

13. Toyoda, S. *et al.* Effect of chemical treatment on ESR dosimetry of cow teeth: Application to the samples from Southern Urals. *Radiation Measurements* **42**, 1178–1180 (2007).

14. Duval, M., Grün, R., Falguères, C., Bahain, J.-J. & Dolo, J.-M. ESR dating of Lower Pleistocene fossil teeth: Limits of the single saturating exponential (SSE) function for the equivalent dose determination. *Radiation Measurements* **44**, 477–482 (2009).

15. Duval, M. *et al.* Electron spin resonance dating of optically bleached quartz grains from the Middle Palaeolithic site of Cuesta de la Bajada (Spain) using the multiple centres approach. *Quaternary Geochronology* **37**, 82–96 (2017).

16. Duval, M., Guilarte Moreno, V. & Grün, R. ESR dosimetry of fossil enamel: some comments about measurement precision, long-term signal fading and dose–response curve fitting. *Radiation Protection Dosimetry* **157**, 463–476 (2013).

17. Bahain, J.-J. *et al.* ESR and ESR/U-series chronology of the Middle Pleistocene site of Tourville-la-Rivière (Normandy, France) - A multi-laboratory approach. *Quaternary International* S1040618218308796 (2019) doi:10.1016/j.quaint.2019.06.015.

18. Mercier, N. & Falguères, C. Field gamma dose-rate measurement with a NaI(Tl) detector: re-evaluation of the ‘threshold’ technique. **25**, 46 (2007).

19. Yokoyama, Y. & Nguyen, H. V. Direct and non destructive dating of marine sediments, manganese nodules and corals by high resolution gamma-ray spectrometry. in *Isotope marine chemistry* 259–289 (Uchida Rokakuho Tokyo, 1980).

20. Guérin, G., Mercier, N. & Adamiec, G. Dose-rate conversion factors: update. *Ancient TL* **29**, 5–8 (2011).

21. Laurent, M., Falguères, C., Bahain, J. J., Rousseau, L. & Van Vliet Lanoé, B. ESR dating of quartz extracted from quaternary and neogene sediments: method, potential and actual limits. *Quaternary Science Reviews* **17**, 1057–1062 (1998).

22. Brennan, B. J., Lyons, R. G. & Phillips, S. W. Attenuation of alpha particle track dose for spherical grains. *International Journal of Radiation Applications and Instrumentation. Part D. Nuclear Tracks and Radiation Measurements* **18**, 249–253 (1991).

23. Brennan, B. J. Beta doses to spherical grains. *Radiation Measurements* **37**, 299–303 (2003).

24. Grün, R. Au cautionary note: use of ‘water contempt’ and ‘depth for cosmic ray dose rate’ in AGE and DATA programs. *Ancient TL* **12**, 50–51 (1994).

25. Prescott, J. R. & Hutton, J. T. Cosmic ray contributions to dose rates for luminescence and ESR dating: Large depths and long-term time variations. *Radiation Measurements* **23**, 497–500 (1994).

26. Murray, A. S. & Roberts, R. G. Determining the burial time of single grains of quartz using optically stimulated luminescence. *Earth and Planetary Science Letters* **152**, 163–180 (1997).

27. Vandenberghe, D., De Corte, F., Buylaert, J.-P., Kučera, J. & Van den haute, P. On the internal radioactivity in quartz. *Radiation Measurements* **43**, 771–775 (2008).

28. Plagnes, V., Causse, C., Genty, D., Paterne, M. & Blamart, D. A discontinuous climatic record from 187 to 74 ka from a speleothem of the Clamouse Cave (south of France). *Earth and Planetary Science Letters* **201**, 87–103 (2002).

29. Cheddadi, R. *et al.* Similarity of vegetation dynamics during interglacial periods. *Proceedings of the National Academy of Sciences* **102**, 13939–13943 (2005).

30. Méndez-Quintas, E. *et al.* First evidence of an extensive Acheulean large cutting tool accumulation in Europe from Porto Maior (Galicia, Spain). *Scientific Reports* **8**, 3082 (2018).

31. Pons-Branchu, E. *et al.* A geochemical perspective on Parisian urban history based on U–Th dating, laminae counting and yttrium and REE concentrations of recent carbonates in underground aqueducts. *Quaternary Geochronology* **24**, 44–53 (2014).

32. Cheng, H. *et al.* Improvements in 230Th dating, 230Th and 234U half-life values, and U–Th isotopic measurements by multi-collector inductively coupled plasma mass spectrometry. *Earth and Planetary Science Letters* **371–372**, 82–91 (2013).

33. Jaffey, A. H., Flynn, K. F., Glendenin, L. E., Bentley, W. C. & Essling, A. M. Precision Measurement of Half-Lives and Specific Activities of 235U and 238U. *Phys. Rev. C* **4**, 1889–1906 (1971).

34. Lanos, P. & Dufresne, P. *ChronoModel version 2.0 User manual*. (2020).

35. Lanos, P., Philippe, A., Lanos, H. & Dufresne, P. *Chronomodel: Chronological modelling of archaeological data using Bayesian statistics.(version 1.5)*. (2016).

36. Lanos, P. & Philippe, A. Hierarchical Bayesian modeling for combining Dates in archaeological context. (2015).

37. Harmand, D. *et al.* Relationships between fluvial evolution and karstification related to climatic, tectonic and eustatic forcing in temperate regions. *Quaternary Science Reviews* **166**, 38–56 (2017).

38. Robert, T. *et al.* Assessing the contribution of electrical resistivity tomography (ERT) and self-potential (SP) methods for a water well drilling program in fractured/karstified limestones. *Journal of Applied Geophysics* **75**, 42–53 (2011).
